# IgA vasculitis in adults: the performance of the EULAR/PRINTO/PRES classification criteria in adults

**DOI:** 10.1186/s13075-016-0959-4

**Published:** 2016-03-02

**Authors:** Alojzija Hočevar, Ziga Rotar, Vesna Jurčić, Jože Pižem, Saša Čučnik, Alenka Vizjak, Rianne van den Broeke, Matija Tomšič

**Affiliations:** Department of Rheumatology, University Medical Centre Ljubljana, Vodnikova cesta 62, 1000 Ljubljana, Slovenia; Institute of Pathology, Faculty of Medicine, University of Ljubljana, Vrazov trg 2, 1104 Ljubljana, Slovenia; Institute for Biostatistics and Medical Informatics, Faculty of Medicine, University of Ljubljana, Vrazov trg 2, 1104 Ljubljana, Slovenia; Faculty of Medicine, University of Ljubljana, Vrazov trg 2, 1104 Ljubljana, Slovenia

**Keywords:** IgA vasculitis, Henoch-Schönlein purpura, Classification criteria

## Abstract

**Background:**

In 2010, EULAR/PRINTO/PRES proposed new classification criteria for paediatric IgA vasculitis (IgAV) that have a higher diagnostic sensitivity than the 1990 ACR criteria. These criteria have so far not been evaluated in adults, in whom IgAV is considered as a rare disease. Our main objective was to compare the diagnostic performance of EULAR/PRINTO/PRES and ACR classification criteria in adult IgAV.

**Methods:**

Adult IgAV cases fulfilling the 2012 revised International Chapel Hill Consensus Conference Nomenclature of Vasculitides (ICHCCNV) definition of IgAV at a secondary/tertiary rheumatology referral centre were critically reviewed in a partially retrospective and partially prospective manner. First, we compared the diagnostic sensitivity of ACR and EULAR/PRINTO/PRES criteria in this group of patients. Second, the diagnostic specificity of ACR and EULAR/PRINTO/PRES was determined by applying these criteria to a control group of patients with other systemic vasculitides.

**Results:**

Between 1 January 2010 and 31 December 2014 350 new cases of systemic vasculitis were identified. IgAV was diagnosed in 129, and other systemic vasculitides in 221 (123 had large, six medium and 92 small vessel vasculitis) cases according to ICHCCNV. The diagnostic sensitivity and specificity of the IgAV EULAR/PRINTO/PRES criteria were 99.2 % (95 % CI 95.4–99.9 %) and 86.0 % (95 % CI 80.7–90.3 %), and of the ACR criteria 86.8 % (95 % CI 79.7–92.1 %) and 81.0 % (95 % CI 75.2–85.9 %), respectively with an inter-criteria agreement of 77.5 % (95 % CI: 70.8–84.1 %).

**Conclusions:**

In the adult population the EULAR/PRINTO/PRES IgAV classification criteria had a higher sensitivity and specificity than the ACR criteria.

## Background

Immunoglobulin A-associated vasculitis (IgAV; formerly known as Henoch-Schönlein purpura) is a leucocytoclastic, immune complex-mediated, small vessel vasculitis characterized clinically by palpable purpura, joint, gastrointestinal, or renal involvement and histologically by the predominance of IgA deposits in the vascular wall. IgAV is regarded as a disease of childhood. However, we have recently shown that IgAV, contrary to common belief, is by no means rare in adults. Our findings suggest a 3–6 times higher incidence rate of adult IgAV than previously reported [1].

The diagnosis of IgAV is based on the signs, symptoms, and histopathological findings. European League Against Rheumatism/Paediatric Rheumatology International Trials Organisation/Paediatric Rheumatology European Society (EULAR/PRINTO/PRES) recently published new classification criteria for childhood vasculitides, including IgAV. In children, IgAV criteria performed better than the older American College of Rheumatology (ACR) classification criteria (Table 1) [2, 3]. A recent Spanish study reported a 52.3 % concordance between the EULAR/PRINTO/PRES and the ACR IgAV criteria in a mixed paediatric and adult historic cohort [4].

**Table 1:** Classification criteria for IgA vasculitis

ACR classification criteria (1990) [2]	EULAR/PRINTO/PRES classification criteria (2010) [3]
Two of the following criteria: • age ≤ 20 years • palpable purpura • acute abdominal pain • biopsy showing granulocytes in the walls of the small arterioles or venules	• Purpura or petechiae AND • One of the following four criteria ◦ abdominal pain ◦ arthritis or arthralgia ◦ renal involvement ◦ leucocytoclastic vasculitis with predominant IgA deposits or proliferative glomerulonephritis with predominant IgA deposits
*sensitivity 87.1 %; specificity 87.7 %*	*sensitivity 100 %; specificity 87 %*

*IgA* immunoglobulin A-associated, *ACR* American College of Rheumatology, *EULAR/PRINTO/PRES* European League Against Rheumatism/Paediatric Rheumatology International Trials Organisation/Paediatric Rheumatology European Society

Thus far the performance of the EULAR/PRINTO/PRES criteria not been evaluated in an exclusively adult population. Our main objective was to compare the diagnostic sensitivity and specificity of the EULAR/PRINTO/PRES and the ACR IgAV classification criteria in adults.

## Methods

### Setting

This partially retrospective (from 1 January 2010 to 31 December 2012), partially prospective (from 1 January 2013 to 31 December 2014) observational study was performed at the Department of Rheumatology, University Medical Centre Ljubljana, Slovenia. The University Medical Centre Ljubljana is an integrated teaching hospital, serving approximately 1,060,000 adult residents at the tertiary level, and is the only secondary level referral hospital in the Ljubljana region, serving approximately 530,000 adult residents.

### Patients and diagnostic criteria

Adults, i.e. persons aged ≥18 years, with systemic vasculitis, diagnosed for the first time during the observation period, were included. Cases fulfilling the 2012 revised International Chapel Hill Consensus Conference Nomenclature of Vasculitides (ICHCCNV) definition of IgAV represented the studied cohort. ICHCCNV and 1990 ACR classification criteria for systemic vasculitides were used to classify patients with giant cell arterits (GCA), Takayasu arteritis, polyarteritis nodosa (PAN), ANCA-associated vasculitides (AAV), and Cogan syndrome [2, 5–9]. The International Study Group for Behçet’s Disease criteria were used for Behçet syndrome, [10] preliminary classification criteria for the cryoglobulinaemic vasculitis, [11] and criteria proposed by Calabrese for diagnosing primary angiitis of the central nervous system [12].

For the retrospective part of the study, cases with vasculitis were ascertained by searching the electronic medical records for the 10th revision of the International Statistical Classification of Diseases code for the particular vasculitis.

Patients with systemic vasculitis other than IgAV served as controls. We used both the 1990 ACR and the 2010 EULAR/PRINTO/PRES classification criteria (Table 1) in our cohort of IgAV patients and controls.

### Diagnostic work-up

After a thorough clinical evaluation, patients underwent an extensive laboratory work-up in accordance with the established local vasculitis management protocol. Markers of systemic inflammation [erythrocyte sedimentation rate (ESR), C-reactive protein (CRP)], a complete blood count with differential, basic biochemistry panels including electrolytes, creatinine, urea, liver function tests, muscle enzymes, serum electrophoresis, urine analysis were done in all patients. Immunoserological screening routinely performed in case of vasculitis included Hep-2 test, antibodies against extractable nuclear antigens (Sm, U1RNP, Ro, La, Scl-70, Jo-1, PCNA, PM/Scl, SL, Ku), rheumatoid factor; anti-neutrophil cytoplasmic antibodies (ANCA) against myeloperoxidase (anti-MPO) and proteinase 3 (anti-PR3); cryoglobulins, C3c and C4. When appropriate (e.g. in case of thrombotic complications), antiphospholipid antibodies (anticardiolipin antibodies, anti-beta-2-glycoprotein I antibodies and lupus anticoagulants) were also determined.

All tissue biopsies, except those of the temporal arteries, were evaluated using both bright field microscopy to confirm leucocytoclastic vasculitis or glomerulonephritis and direct immunofluorescence to demonstrate IgA or other immunoglobulin (IgM, IgG), fibrin/fibrinogen and complement deposits.

The use of imaging, endoscopy and sub-speciality consultations was guided by the clinical picture.

### Statistical analysis

Diagnostic sensitivity, specificity and the inter-criteria agreement Cohen’s kappa coefficient of EULAR/PRINTO/PRES and ACR IgAV criteria were determined. Comparisons between classification criteria were done using the mid-P variant of McNemar’s test. InteractiVenn online software was used to draw the Venn diagrams [13].

### Patient consent and ethics committee approval

Patient consent was not needed since the collected data stems from routine clinical practice. The study was approved by the Slovenian National medical ethics committee.

## Results

During the 60-month observation period, between 1 January 2010 and 31 December 2014, 350 new patients with vasculitis were identified, including 129 IgAV cases (67 retrospectively and 62 prospectively) (Table 2).

**Table 2:** Vasculitides diagnosed between January 2010 and December 2014

		Classified as IgAV by	
	All (N)	EULAR/PRINTO/PRES criteria (N)	ACR criteria (N)
IgA vasculitis	129	128	112
Controls	221	31	42
Giant cell arteritis	121	0	0
ANCA-associated vasculitis	38	13	11
Cryoglobulinaemic vasculitis	18	10	14
Secondary vasculitis	11	5	8
Single organ vasculitis	8	0	8
Behçet syndrome	7	0	0
Polyarteritis nodosa	6	0	0
Takayasu arteritis	2	0	0
Cogan syndrome	2	0	0
Anti-GBM disease	1	0	0
Primary angiitis of the central nervous system	1	0	0
Anti-C1q vasculitis	1	0	0
Unclassified vasculitis	5	3	1

*IgAV* immunoglobulin A-associated vasculitis, *EULAR/PRINTO/PRES* European League Against Rheumatism/Paediatric Rheumatology International Trials Organisation/Paediatric Rheumatology European Society, *ACR* American College of Rheumatology, *ANCA* anti-neutrophil cytoplasmic antibodies, *GBM* glomerular basement membrane

Fifty-eight percent of the IgAV patients were male. The median age at the time of presentation was 64.2 years [range 18–92, interquartile range (IQR) 40.4–77.3]. Four patients were younger than 21 years. Palpable purpura was present in all patients. Fifty-eight (45 %) patients had joint involvement. Gastrointestinal (GI) involvement was present in 48 (37.2 %) patients and kidneys were affected in 65 (50.4 %) patients. All IgAV patients underwent a skin biopsy. Leucocytoclastic vasculitis was present in 124/129 (96.1 %) patients. In five patients only perivascular infiltration was seen. Granulocytes in vessel wall were found in 106 (82.2 %) cases. IgA-dominant immune vascular deposits were demonstrated in the tissue biopsy specimens from all IgAV patients.

Our control group consisted of 221 cases with vasculitis other than IgAV diagnosed during the same time period. Large vessel vasculitis was diagnosed in 123 cases, medium vessel vasculitis in six and small vessel vasculitis in 92 cases (Table 2).

### Comparison of classification criteria

**Fig. 1:**
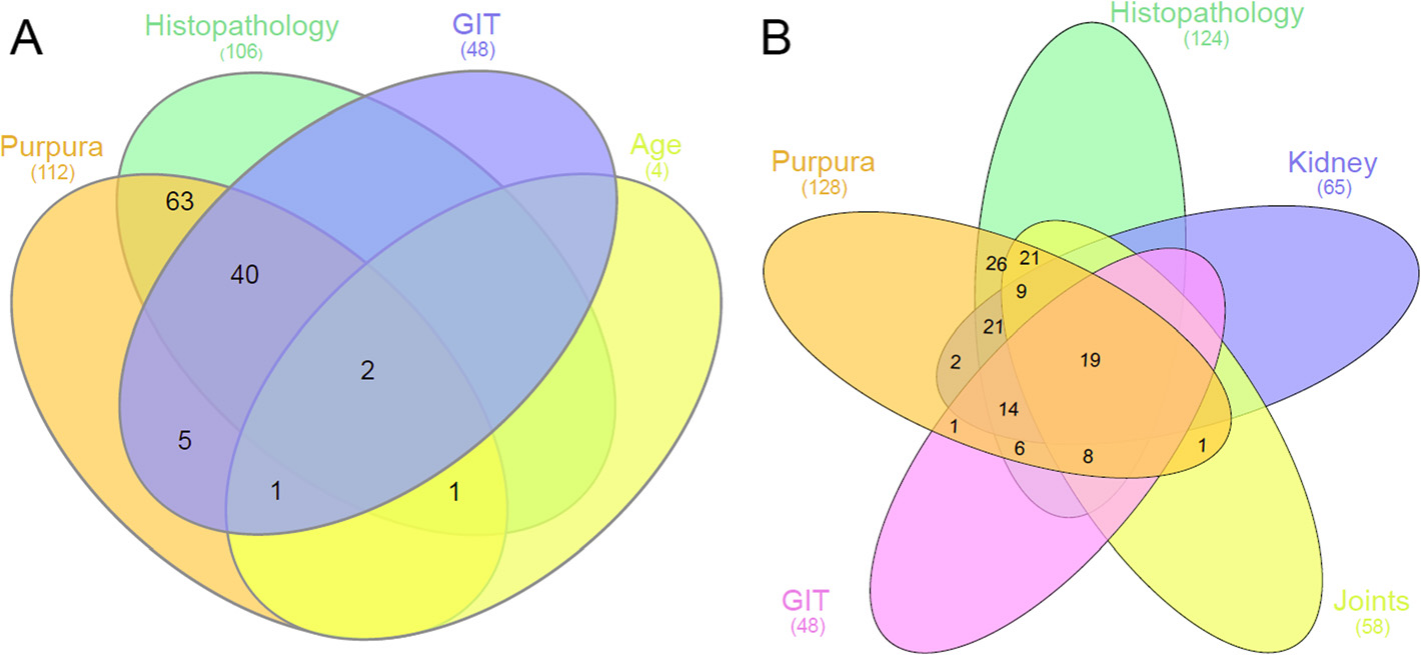
Venn diagram of the classification criteria items fulfilled during the acute phase of the disease. **a** American College of Rheumatology criteria (1990). **b** European League Against Rheumatism/Paediatric Rheumatology International Trials Organisation/Paediatric Rheumatology European Society criteria (2010)

In the IgAV cohort, 112/129 (86.8 %) patients fulfilled ACR, and 128 (99.2 %) patients EULAR/PRINTO/PRES IgAV criteria. The criteria fulfilled in the cases classified by either criteria are depicted in the Venn diagrams (Fig. 1).

In the control group, 31/221 (14.0 %) patients could also be classified as IgAV using EULAR/PRINTO/PRES, and 42/221 (19.0 %) by ACR IgAV criteria. Twenty-five controls (11.3 %) fulfilled both IgAV classification criteria. Six (2.7 %) controls fulfilled exclusively EULAR/PRINTO/PRES and 17 (7.7 %) controls exclusively ACR IgAV criteria. Twenty-nine out of 31 (93.5 %) controls that fulfilled the EULAR/PRINTO/PRES IgAV classification criteria, did so by clinical items (i.e. purpura plus joint and/or GI tract and/or kidney involvement). Two cases, classified as cryoglobulinaemic vasculitis based on a history of recurrent episodes of purpura, nephritis and in one case a peripheral neuropathy, the presence of cryoglobulins, low C4, and disease evolution on follow-up, additionally fulfilled the histological item of the EULAR/PRINTO/PRES criteria – i.e. the demonstration of predominant IgA immunoglobulin deposits on the skin biopsy.

The sensitivity and specificity of IgA-dominant vascular deposits in the case of small vessel vasculitis for IgAV were 100 % (95 % CI: 97–100 %) and 97.8 % (95 % CI: 92.3–99.7 %), respectively with a positive predictive value of 98.5 % (95 % CI: 94.6–99.8 %).

The controls that fulfilled the ACR IgAV criteria did so on the basis of clinical items in 4/42 cases only. The remaining 38 cases met the ACR criteria by the combination of clinical and histological items.

The diagnostic sensitivity and specificity of the EULAR/PRINTO/PRES vs the ACR IgAV criteria were 99.2 % (95 % CI: 95.4–99.9 %) vs 86.8 % (95 % CI: 79.7–92.1 %) (*p* = 0.02) and 86.0 % (95 % CI: 80.7–90.3 %) vs 81.0 % (95 % CI: 75.2–85.9 %) (*p* < 0.01), respectively. The positive and negative likelihood ratios of the EULAR/PRINTO/PRES vs the ACR IgAV criteria were 7.07 (95 % CI: 5.10–9.81) vs 4.57 (95 % CI: 3.45–6.05) and 0.01 (95 % CI: 0.00–0.06) vs 0.16 (95 % CI: 0.10–0.25), respectively. The inter-criteria agreement Cohen’s kappa coefficient was 77.5 % (95 % CI 70.8–84.1 %).

## Discussion

In the absence of diagnostic criteria for the systemic vasculitides, clinicians often rely on the existing classification criteria when faced with a patient presenting with kaleidoscopic symptoms and signs suggestive of systemic vasculitis to make the final diagnosis. Early and correct diagnosis is of paramount importance for favourable outcomes. A glimpse of the recent new insights into these complex diseases is reflected in the ICHCCNV, which has not yet been followed by an update of the classification criteria [6].

In the case of IgA vasculitis we usually rely on the 1990 ACR IgAV classification criteria. They were based on the data analysis from 807 patients with systemic vasculitides, 85 of them were classified as having IgAV [2]. In 2010, the Pediatric Rheumatology European Society (PRES) endorsed by the EULAR published a new set of classification criteria for paediatric vasculitides, including the IgAV. The EULAR/PRINTO/PRES IgAV classification criteria are based on data analysis from 827 children with IgAV. In children, the newer EULAR/PRINTO/PRES criteria have a higher diagnostic sensitivity than the older ACR criteria without any loss of diagnostic specificity (Table 1) [3].

This is the first report evaluating the performance of the new EULAR/PRINTO/PRES criteria in an exclusively adult cohort. Our data suggests that, like in the paediatric population, the EULAR/PRINTO/PRES are more sensitive than the older ACR IgAV criteria in the adult population. The two main reasons for the increased sensitivity of the EULAR/PRINTO/PRES are the addition of joint and kidney involvement, and the modification of the histopathological criterion by the addition of the IgA vessel wall deposition. Considering only the clinical items of both criteria sets, diagnosis of IgAV could be made in about 80 % and 40 % of cases if the EULAR/PRINTO/PRES and ACR criteria were used, respectively. While all our patients had purpura with lower limb predominance, the clinical items of the ACR criteria were only fulfilled by four patients younger than 21 years, and 48 of those with GI involvement. Although the sensitivity of the ACR criteria improved when we considered the histopathology, it still lagged behind that of the EULAR/PRINTO/PRES criteria. In our cohort the ACR histological criterion, requiring granulocyte infiltration of the vessel wall, was not met in 23 (17.8 %) of IgAV patients, while leucocytoclastic vasculitis with IgA deposits was found in 124 (96.1 %) IgAV patients and lone IgA vascular deposits in five cases. The reason for the inability to prove vasculitis in some cases might rest in the fact that fresh skin lesions were not always obtainable. In these cases, perivascular inflammatory infiltrates and the demonstration of IgA deposits in the vessel walls implied IgAV. Considering all four ACR criteria items, 17 (13.2 %) patients from our IgAV cohort did not meet the classification criteria, compared to a single one (0.8 %) when using the EULAR/PRINTO/PRES criteria (Fig. 1). The EULAR/PRINTO/PRES criteria also have a significantly higher diagnostic specificity than the ACR IgAV criteria (86.0 % vs 81.0 %, *p* = 0.023). The validity of our results is supported by the recent Spanish study, which suggested the applicability of EULAR/PRINTO/PRES IgAV criteria and showed that these criteria were fulfilled more often than ACR or Michel criteria in the adult subset of the mixed paediatric and adult cohort of patients with primary cutaneous vasculitis. However, they only found a 52.3 % concordance between the EULAR/PRINTO/PRES and ACR IgAV criteria [4]. The majority (93.5 %) of controls were misclassified as IgAV when using the EULAR/PRINTO/PRES criteria based on the clinical items. Only two controls were misclassified as IgAV by the demonstration of IgA-dominant immunoglobulin deposits on the skin biopsy. In contrast, more than 95 % of the controls misclassified as IgAV when using ACR criteria also fulfilled the histological criterion. The demonstration of IgA deposits, required by the EULAR/PRINTO/PRES criteria, significantly narrows down the differential diagnosis of the leucocytoclastic small vessel vasculitis and enhances the diagnostic specificity of these criteria. Our observation is in disparity with Larson who reported an 86 % diagnostic sensitivity and an 84 % specificity of IgA deposits [14].

The most obvious limitations of our study are that it was conducted in a single secondary/tertiary centre making it subject to a channelling bias, its partly retrospective nature, and the rather small sample size. Yet we feel that the results of the first study comparing the EULAR/PRINTO/PRES and ACR IgAV criteria are compelling due to the thorough systematic clinical and laboratory work-up of patients suspected of having systemic vasculitis and the inclusion of histologically proven IgAV cases.

## Conclusions

The use of the EULAR/PRINTO/PRES criteria in the adult population seems feasible, given that they are more sensitive and specific than the 1990 ACR IgAV criteria.

## Abbreviations

AAV: ANCA-associated vasculitides
ACR: American College of Rheumatology
ANCA: Anti-neutrophil cytoplasmic antibodies
CRP: C-reactive protein
ESR: Erythrocyte sedimentation rate
EULAR/PRINTO/PRES: European League Against Rheumatism/Paediatric Rheumatology International Trials Organisation/Paediatric Rheumatology European Society
GCA: Giant cell arterits
GI: Gastrointestinal
ICHCCNV: International Chapel Hill Consensus Conference Nomenclature of Vasculitides
IgAV: Immunoglobulin A-associated vasculitis
IQR: Interquartile range
MPO: Myeloperoxidase
PAN: Polyarteritis nodosa
PR3: Proteinase 3
